# Arthroscopic Anterior Cruciate Ligament Repair With Single-Bundle Hamstring Tendon Augmentation Providing Complete Femoral Footprint Restoration for Sherman Type One Tears: A Technique Guide

**DOI:** 10.1016/j.eats.2022.07.009

**Published:** 2022-10-20

**Authors:** Rubén Monárrez, Craig Bennett

**Affiliations:** Rubin Institute for Advanced Orthopedics, Sports Medicine, Sinai Hospital, Baltimore, Maryland, U.S.A.

## Abstract

There is renewed interest in performing arthroscopic anterior cruciate ligament (ACL) repairs in appropriate patients who have Sherman type 1 ACL tears. However, ACL repairs are associated with unacceptably high failure rates, which may be partly improved with suture augmentation. Our technique uses a hamstring autograft tendon to reconstruct a bundle through a femoral tunnel while inserting the native ACL tissue into the other bundle’s femoral footprint. The tear pattern dictates whether the native ACL tissue is inserted into the anteromedial or posterolateral lateral femoral origin. The improved cellular and biomechanical milieu for healing of both the repair and reconstruction may translate to earlier return to sport and reduced failure rates. In addition, by restoring the entire femoral footprint with a single femoral tunnel, improved rotational control is achieved without the bone stock loss observed in traditional ACL double-bundle reconstruction.

In recent years, there has been renewed interest in preforming anterior cruciate ligament (ACL) repair in select patients who have proximal primary ACL avulsion tears known as Sherman type 1.^1^ Unfortunately, repair of Sherman type 1 tears in patients aged 21 years or younger has been associated with an unacceptably high failure rate.^2^ Vermeijden et al.^3^ have published an innovative method of augmenting an ACL repair using a single-bundle graft. In their proposed technique, one bundle is repaired and one bundle is reconstructed with an allograft through a single femoral tunnel using extracortical fixation.

While it remains unclear whether anatomic autograft double-bundle (db) anterior cruciate ligament reconstruction (ACLR) reduces failure rates when compared with single-bundle ACLR, the lead author uses dbACLR as his technique of choice for high-risk patients including female athletes, those with baseline increased ligamentous laxity, and those with large ACL insertion footprints,^4^, ^5^, ^6^ Reconstructing the entire ACL footprint with a dbACLR is associated with a larger cross-sectional area of the ACL graft and may also improve dynamic rotational laxity.^7^, ^8^, ^9^ We hypothesized that by restoring the entire femoral footprint in an ACL repair with single-bundle autograft augmentation, there would be a reduction in failure rates and reoperation rates when compared with ACL repair alone and an earlier return to sport when compared with ACLR.

## Surgical Technique (With Video Illustration)

### Patient Selection

Patients are selected and offered a primary ACL repair with single-bundle autograft augmentation if they have a native ACL Sherman type 1 tear pattern diagnosed on magnetic resonance imaging, have full preoperative range of motion, are ready for surgery within 4 weeks of injury, and are skeletally mature. Intraoperatively, tear pattern and tissue quality are evaluated arthroscopically and if felt to be of poor-quality patients are instead treated with ACLR.

### Surgical Procedure

**Fig 1:**
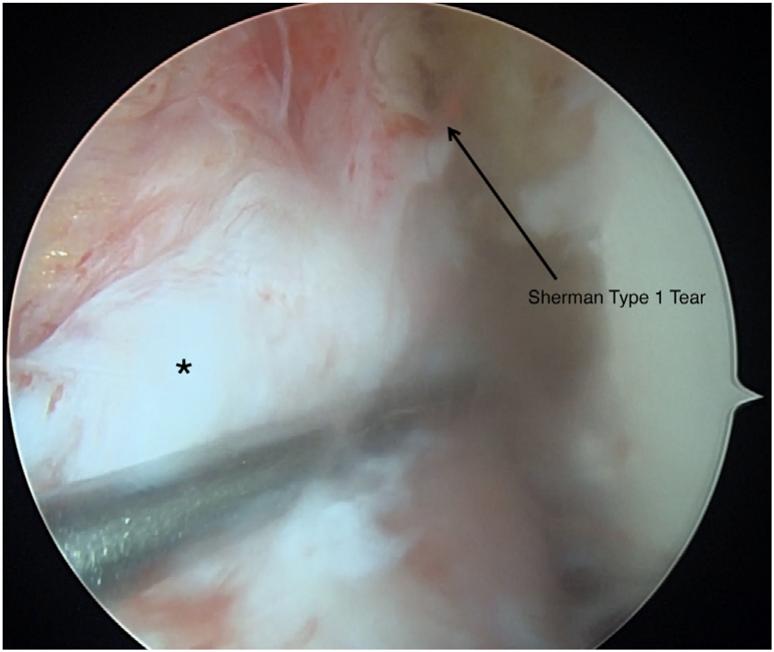
Arthroscopic view of a left knee, viewed from an anterolateral portal with the patient supine and the knee at 90° of flexion. A Sherman type 1 ACL tear (arrow) with excellent tissue quality (asterisk) is shown. This tear pattern was determined to be a long ACL stump and will therefore be reinserted into the anteromedial ACL femoral footprint to provide adequate tension. (ACL, anterior cruciate ligament.)

**Fig 2:**
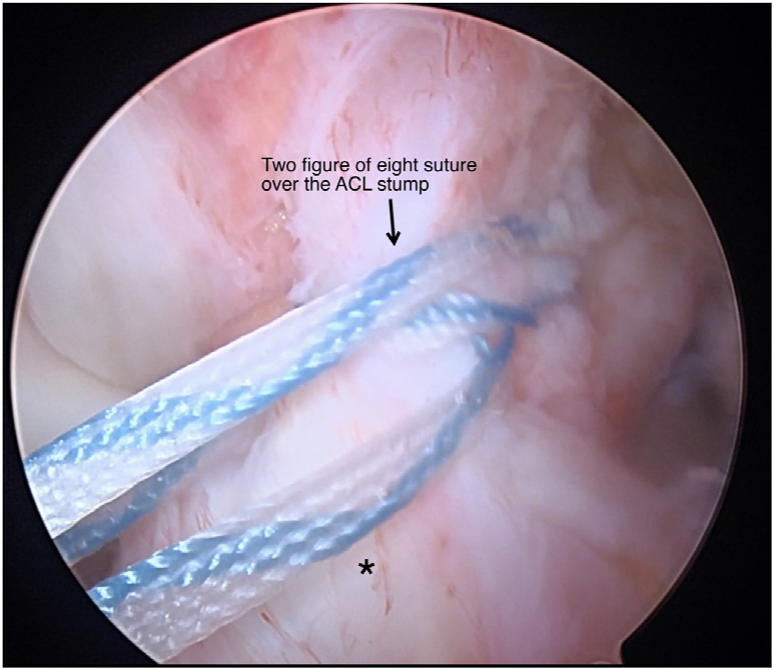
Arthroscopic view of a left knee, viewed from an anterolateral portal with the patient supine and the knee at 90° of flexion. Two figure of eight sutures (arrow) over the mid- and mid-proximal ACL stump (asterisk). This will be reinserted into the femoral footprint. (ACL, anterior cruciate ligament.)

**Fig 3:**
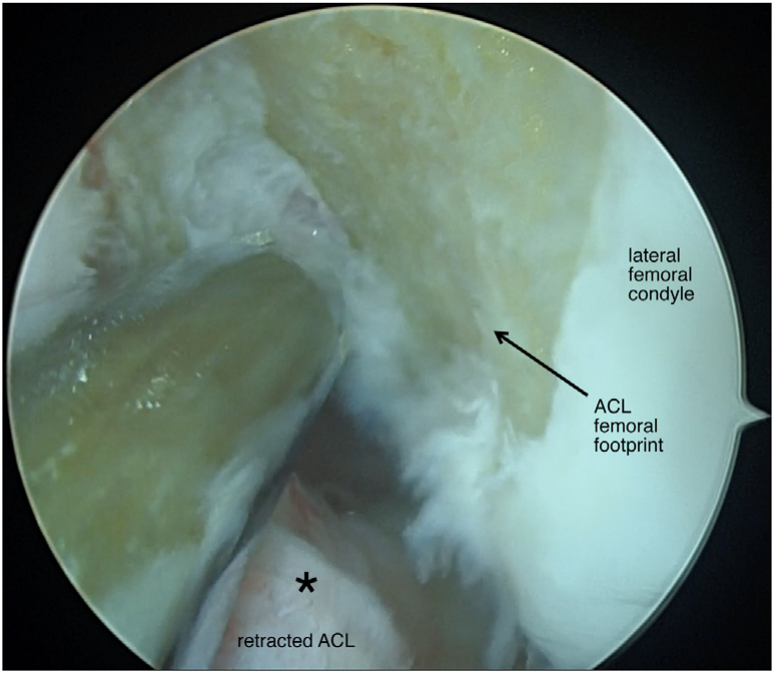
Arthroscopic view of a left knee, viewed from an anterolateral portal with the patient supine and the knee at 90° of flexion. The femoral footprint (arrow) of the ACL is prepared with a shaver (Arthrex, Naples, FL). The native ACL tissue (asterisk) is retracted to avoid injury during preparation of the femoral footprint. (ACL, anterior cruciate ligament.)

**Fig 4:**
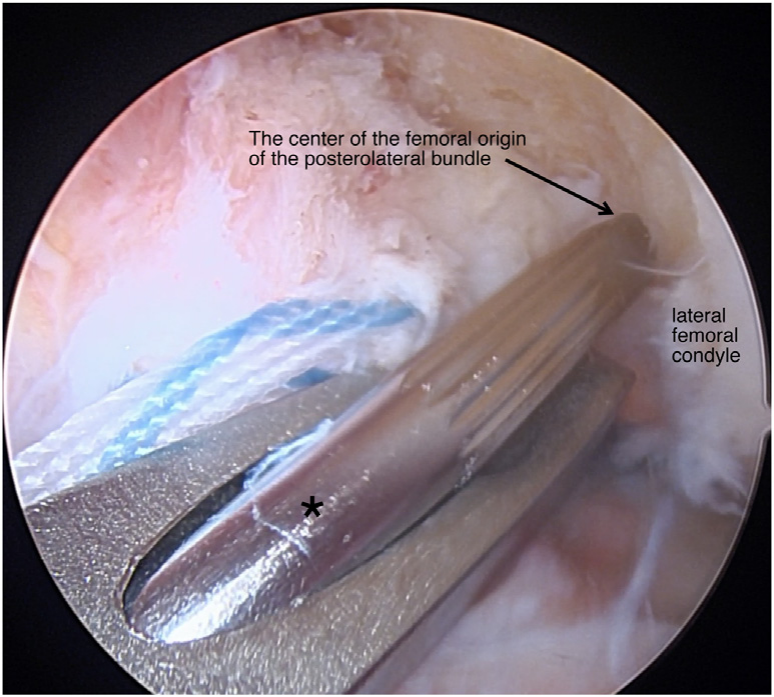
Arthroscopic view of a left knee, viewed from an anterolateral portal with the patient supine and the knee at 90° of flexion. A Beath pin (asterisk) is drilled though the center of the femoral origin (arrow) of the posterolateral bundle using an accessory medial portal. The posterolateral bundle will be reconstructed with an allograft.

**Fig 5:**
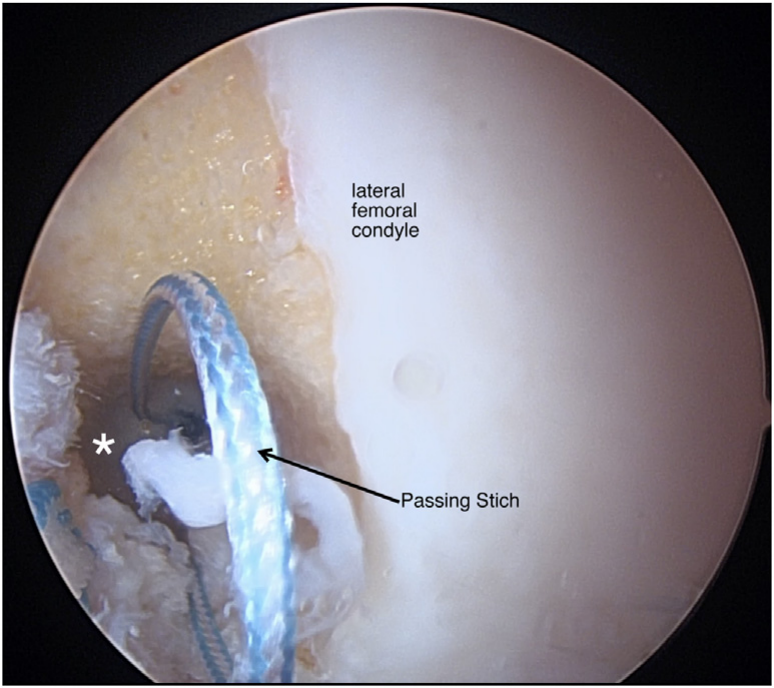
Arthroscopic view of a left knee, viewed from an accessory medial portal with the patient supine and the knee at 90° of flexion. A passing stitch (arrow) is placed through the femoral socket (asterisk) after the femoral tunnel is dilated and cleaned of bony debris. This will be used to tunnel the allograft to reconstruct the posterolateral bundle.

Patients are placed in the supine position and the operative leg is prepped and draped in a sterile fashion (Table 1 and Video 1). Standard anteromedial and anterolateral portals are made and a 30° scope (Arthrex, Naples, FL) is introduced through the anterolateral portal. A systemic diagnostic evaluation is then performed and the ACL tear pattern and tissue quality are evaluated (Fig 1). Once the patient is confirmed to be an appropriate candidate for an ACL repair, 2 SutureTape (Arthrex, Naples, FL) sutures in a figure-of-eight technique are placed into the mid- and mid-proximal portions of the avulsed ACL using a Scorpion suture passer (Arthrex) (Fig 2). The SutureTape (Arthrex) sutured to the ACL stump is then brought through the medial portal to prevent injury to the native ACL and improve visualization during the single-bundle reconstruction. The footprint of the anteromedial (AM) bundle between the 1- to 2-o’clock position (left knee) to be inserted is then lightly debrided with a shaver (Fig 3). The tear pattern dictates whether the native ACL tissue is inserted into the AM or posterolateral (PL) lateral femoral origin. To reinsert the repaired bundle under appropriate tension into the femoral footprint, the AM bundle (which averages 32 mm in length^10^) origin is chosen in tears with long ACL stumps. In tears with shorter ACL stumps, the PL bundle (which averages 17.8 mm in length^10^) origin is selected to reinsert the native ACL tissue into. Attention is then turned to the PL ACL bundle femoral footprint. A 6-mm over-the-top guide (Arthrex) is placed through the center of the femoral origin of the PL bundle at the 3-o’clock position with the knee at 100° to 110° of flexion using an accessory medial portal (Fig 4). Using the guide, a transfemoral Beath pin (Arthrex) is drilled through the femur and then overdrilled with an appropriately sized (predetermined by the diameter of the graft) low-profile drill bit and is advanced to a depth of 25 to 30 mm through the intercondylar notch of the femur. The femoral tunnel is then dilated and the cortical bone is opened with a 4.5-mm drill bit. After removal of bony debris with a shaver (Arthrex), a passing stitch is placed in the femoral socket (Fig 5).

**Table 1:** Surgical Pearls and Pitfalls Arthroscopic ACL Repair with Single-Bundle Tendon Augmentation

Pearls	Pitfalls
Assess the ACL tear intraoperatively and be equipped to convert to an ACLR if the tissue quality is poor.	Injury to the native ACL tissue during tibial and femoral tunnel preparation.
Use an accessory medial portal to optimize drilling the femoral tunnels and reinsertion of the repaired bundle.	Overtensioning or undertensioning of native ACL tissue as it’s inserted into the femoral footprint.
Protect the native ACL tissue by hand reaming the tibial tunnel and using a curette.	
Use an InternalBrace (Arthrex) alongside the autograft for added mechanical support.	

ACL, anterior cruciate ligament; ACLR, anterior cruciate ligament reconstruction.

**Fig 6:**
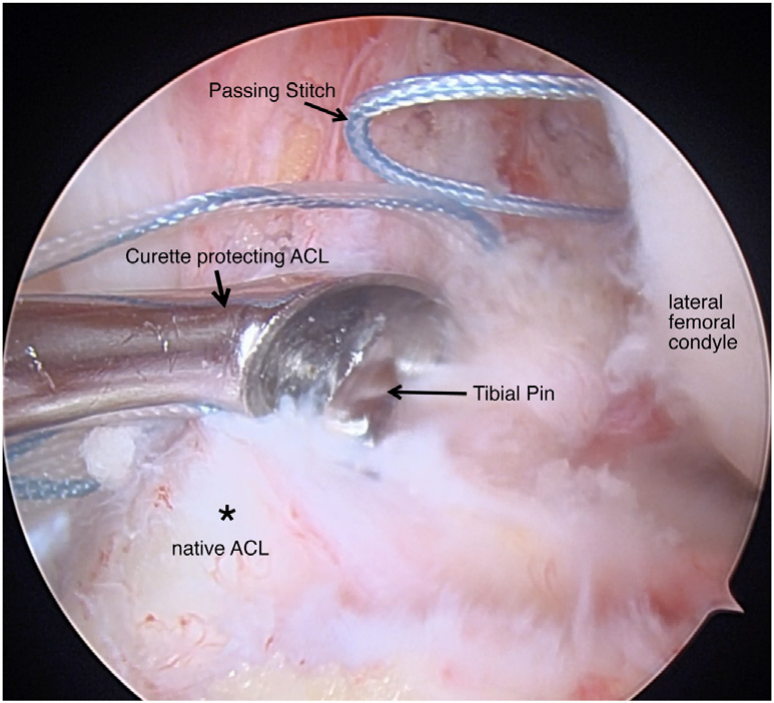
Arthroscopic view of a left knee, viewed from an anterolateral portal with the patient supine and the knee at 90° of flexion. A tibial pin (thin arrow) is advanced through the posterolateral ACL footprint by hand and with a curette (thick arrow) to prevent injury to the ACL stump (asterisk). (ACL, anterior cruciate ligament.)

**Fig 7:**
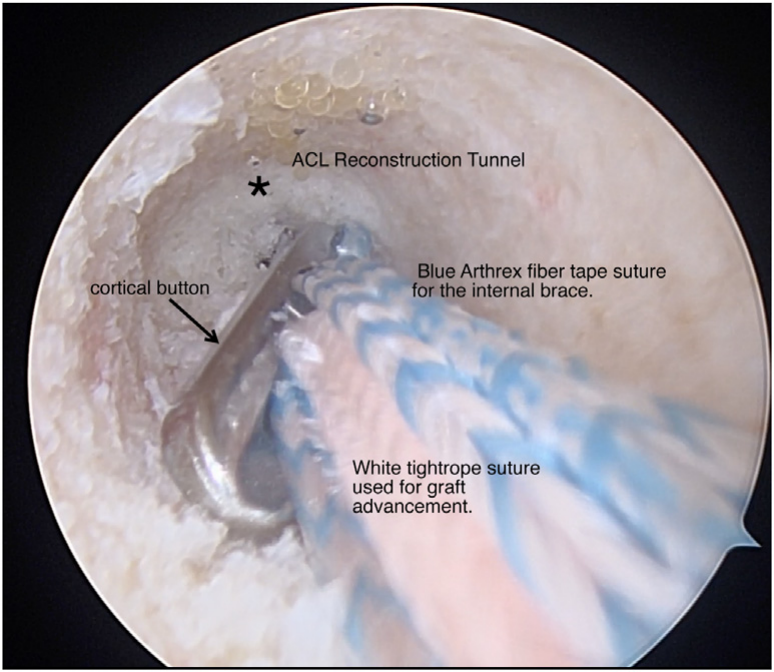
Arthroscopic view of a left knee, viewed from an anterolateral portal with the patient supine and the knee at 90° of flexion. A graft is passed through the tibial tunnel and up the femoral ACL reconstruction tunnel (asterisk) using a cortical button (arrow). This represents the reconstructed posterolateral bundle. (ACL, anterior cruciate ligament.)

The tibial side is then prepared with the 30° arthroscope on the anterolateral portal. An ACL tibial guide is centered over the PL tibial footprint of the tunnel to be reconstructed. A tibial pin (Arthrex) is advanced through the tibia at 55° of flexion and overdrilled using and appropriately sized low-profile drill bit determined by the dimeter of the graft (Fig 6). Care is taken to not violate the intact fibers of the tibial insertion of the bundle to be repaired and, if needed, these tunnels are hand drilled. The tibial tunnel is then dilated. Using the passing stich previously placed though the femoral socket, the sutures whipstiched to the autograft hamstring graft are brought through the tibial tunnel up the femoral tunnel. The graft is then advanced up the transtibial tunnel and under medial portal visualization, the cortical button is flipped and 25 to 30 mm of graft is advanced up the femoral tunnel by shortening the cinch sutures (Fig 7).

**Fig 8:**
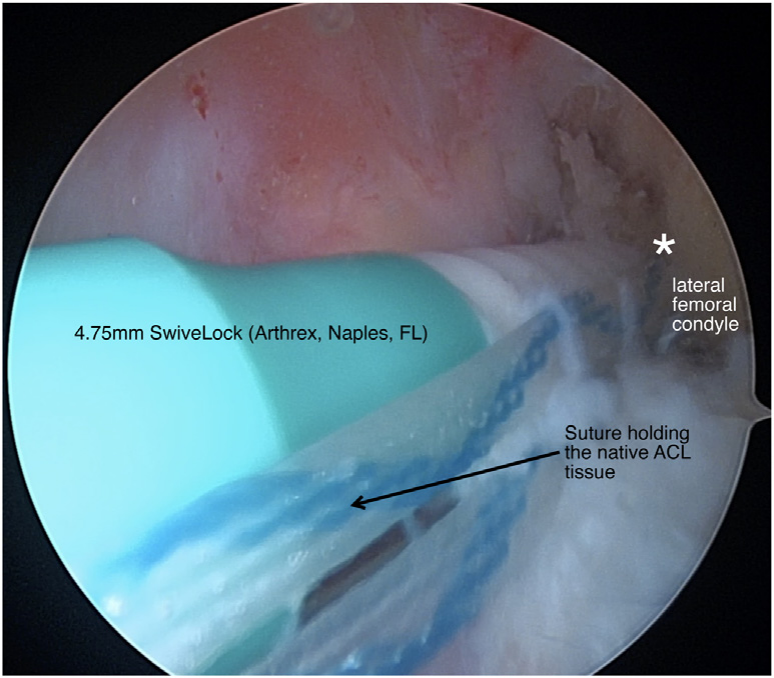
Arthroscopic view of a left knee, viewed from an anterolateral portal with the patient supine and the knee at 90° of flexion. A 4.75-mm SwiveLock (Arthrex, Naples, FL) is used to insert the native ACL tissue (arrow) into the anteromedial ACL bundle femoral origin (asterisk) through the same accessory anteromedial portal used to drill the femoral tunnel. This represents the reinserted native anteromedial ACL bundle. (ACL, anterior cruciate ligament.)

The SutureTape securing the native ACL tissue are then brought through the medial portal and attached to a 4.75-mm SwiveLock (Arthrex). With the knee at 90° of flexion, the native ACL tissue is inserted to the lateral wall at the AM bundle femoral footprint via the SwiveLock (Arthrex) (Fig 8).

The knee is then cycled multiple times and the reconstructed bundle is then tensioned using a BioComposite 23-mm soft tissue interference screw (Arthrex) at 10° of flexion from the patient’s full knee extension while a posterior drawer is applied. The knee is then cycled again and cinch stiches of the proximal TightRope RT (Arthrex) are retightened. The knee is then tested for range of motion and the repaired bundle and reconstructed bundle are assessed for impingement in flexion and extension. The braided sutures are then attached to the distal portion of the graft and secured to the anterior tibia using 4.75-mm SwiveLock (Arthrex) with the knee at 20° of flexion. If an InternalBrace (Arthrex) is used, a few millimeters of tension are taken off the InternalBrace (Arthrex) prior to securing it to the tibia. The retained sutures of the SwiveLock (Arthrex) are then sutured to the graft using a free needle as a third point of tibial-sided fixation. Lachman is then assessed intraoperatively to ensure a grade 0. The surgical wounds are then closed in a standard fashion.

### Graft Harvesting and Preparation

A single tendon (semitendinosus) or 2-tendon (semitendinosus and gracilis) autograft is harvested for the single-bundle reconstruction through a standard anteromedial incision. The grafts are then prepared in a standard fashion and folded over a TightRope RT (Arthrex) with an attached cortical button. The open end of the graft is whipstiched using a #2 FiberWire (Arthrex). An InternalBrace (Arthrex) also can be used alongside the prepared graft. The graft is maintained under tension in the back table.

### Postoperative Management

Patients are placed in a hinged knee brace locked in extension in the operating room. If no meniscal repairs were performed, the patient is encouraged to weight bear as tolerated in extension. Range of motion exercises are begun within the first postoperative week. Once quadriceps control is regained, the brace is unlocked. Absent of meniscal or cartilage surgery, patients are typically cleared to begin a running protocol at 4 months, agility drills at 6 months, and a gradual return to sports is allowed at approximately 7 to 9 months when the patient achieves 90% quadriceps strength compared to the contralateral leg and the patient passes return to play standards.

## Discussion

Mid-term follow-up of arthroscopic ACL repair reveals high failure rate; however, direct comparisons have suggested a lower failure rate in ACL repairs with suture augmentation such as an InternalBrace.^11^ It is theorized, the suture augmentation lowers the ACL repair failure rate by providing mechanical support to the ACL repair as it matures. Our technique uses a hamstring autograft to serve as a biologic internal brace for our ACL repair. Furthermore, in the technique presented in this article, the ACL remanent and repaired ACL bundle provide mechanical reinforcement and cellular migration to the autograft tendon used to reconstruct the PL bundle—allowing graft maturity to proceed more quickly.^12^ The improved cellular and biomechanical milieu for healing of both the repair and reconstruction may translate to earlier return to sport and reduced failure rates.

The clinical relevance of dbACLR remains much debated; however, it has been demonstrated to be associated with a larger cross-sectional area of the ACL graft and also may improve dynamic rotational laxity.^7^, ^8^, ^9^ With this technique, we are restoring the entirety of the femoral footprint with only a single femoral tunnel. Therefore, the technique presented provides the improved rotational control without compromising femoral bone stock in the event a revision is needed in the future (Table 2).

**Table 2:** Advantages and Disadvantages of Arthroscopic ACL Repair with Single-Bundle Tendon Augmentation

Advantage	Disadvantage
Preserves native ACL proprioception.	Indications are limited.
Complete femoral ACL footprint restored providing a large cross-sectional area.	Technically demanding.
Repaired bundle provides mechanical reinforcement and cellular migration to the autograft tendon to promote graft maturity	Difficult to adjust tension of bundle repaired/reinserted.
Reconstructed bundle provides mechanical support to the reinserted bundle.	
Only a single femoral tunnel is drilled to provide the double bundle reconstruction/repair hybrid	
Allows for earlier rehabilitation, range of motion, and return to sport.	

ACL, anterior cruciate ligament.

The use of an autograft hamstring tendon is advocated for in this technique due to the improved cellular and biomechanical milieu described previously—albeit its efficacy when compared with bone–patella–bone in ACLR alone remains contested.^13^ The use of a hamstring tendon autograft diminishes the morbidity associated with bone–patella–bone tendon harvesting; however, semitendinosus harvesting may be associated with reduced postoperative flexion and tibial internal rotation strength.^14^ Future research is needed to determine whether a gracilis-only autograft will suffice for the reconstruction autograft in this technique while avoiding the loss of strength associated with semitendinosus harvesting.^15^

## Supplementary Data

ICMJE author disclosure forms

Video 1 Technique video for arthroscopic anterior cruciate ligament repair with single-bundle hamstring tendon augmentation providing complete femoral footprint restoration for Sherman type one tears.
